# Associations between Familial Factor, Trait Conscientiousness, Gender and the Occurrence of Type 2 Diabetes in Adulthood: Evidence from a British Cohort

**DOI:** 10.1371/journal.pone.0122701

**Published:** 2015-05-06

**Authors:** Helen Cheng, Luke Treglown, Scott Montgomery, Adrian Furnham

**Affiliations:** 1 Department of Psychology, University College London, London WC1E 6BT, United Kingdom; 2 ESRC Centre for Learning and Life Chances in Knowledge Economies and Societies, Institute of Education, University of London, London WC1H 0AL, United Kingdom; 3 Department of Psychology, University of Bath, United Kingdom; 4 Clinical Epidemiology and Biostatistics, Faculty of Medicine and Health, Örebro University, 701 82 Örebro, Sweden; 5 Research Department of Epidemiology and Public Health, UCL, London WC1E 7HB, United Kingdom; 6 BI: Norwegian Business School, Nydalsveien 37, 0484 Oslo, Norway; Universidad Europea de Madrid, SPAIN

## Abstract

**Objective:**

To investigate social, familial, and psychological factors in influencing the occurrence of type 2 diabetes in adulthood.

**Method:**

Some 17,415 babies born in Great Britain in 1958 and followed up at 7, 11, 33, and 50 years of age. The prevalence of type 2 diabetes at age 50 years was the outcome measure.

**Results:**

Some 5,032 participants with data on parental social class, childhood cognitive ability tests scores at age 11 years, educational qualifications at age 33 years, personality traits, occupational levels, and type 2 diabetes (all measured at age 50 years) were included in the study. Available information also included whether cohort members’ parents or siblings had diabetes. Using logistic regression analyses, results showed that sex (OR=0.63: 0.42-0.92, *p*<.05), family history (OR=3.40: 1.76-6.55, *p*<.01), and trait conscientiousness (OR=0.76: 0.64-0.90, *p*<.001) were all significantly and independently associated with the occurrence of type 2 diabetes in adulthood. It appears that the occurrence of type 2 diabetes is greater among men than women (4.3% vs 2.5%).

**Conclusion:**

Familial (genetic and non-genetic) and psychological factors are significantly associated with the prevalence of type 2 diabetes in adulthood.

## Introduction

Research in type 2 diabetes mellitus (T2DM) has shown that this health condition relates to factors such as life style, eating a poor diet, and not exercising [1]. For example, in a sample of 38,018 women aged ≥ 45 years and free of cardiovascular disease, cancer and diabetes with an average 8.8y of follow-up, Song and colleagues [2] found that among flavonoid-rich foods, apple and tea consumption was associated with diabetes risk. Women consuming ≥1 apple/d showed a significant 28% reduced risk of type 2 diabetes compared with those who consumed no apples (the multivariate-adjusted RR = 0.72, 95% CI: 0.56, 0.92; p = 0.006 for trend). Genetic heterogeneity may also be associated with T2DM. However, identifying genetic susceptibility loci has been difficult due to genetic heterogeneity and the context-dependency of disease expression which is strongly related to environmental factors such as eating habits [1].

There is an extensive literature on personality correlates of health and illness. The relationship is correlational not causal and the same biological mechanisms may underlie both; traits lead to behaviours that lead to health differences; illnesses cause personality changes [3].

The area has been recently reviewed by Friedman and Kern [4], indicating that conscientiousness may plays a significant role in health. Keweloh and colleagues [5] investigated the link between personality traits and glycated haemoglobin (HbA_1c_) levels as an indicator of poor blood-sugar level control. They found that T2DM combined with high levels of conscientiousness result in lower levels of HbA_1c_, and thus better blood-sugar level control [5]. Studies also indicate that individuals with high levels of extraversion (particularly the positive emotion facet) have a decreased risk of abnormal glucose regulation [6].

High neuroticism (specifically impulsiveness) and low conscientiousness have been associated with high levels of HDL cholesterol and triglycerides [7], as well as contributing to a larger waist circumference and higher BMI [8]. Jokela and colleagues found that high levels of conscientiousness are linked with lower levels of obesity risk [9], and in turn lower risk of T2DM onset [10]. Shim et al [11] also found that facets of conscientiousness (specifically achievement striving) were associated with lower levels of abnormal glucose regulation. This was the case as these individuals more frequently checked their blood glucose levels than their non-conscientious counterparts. Skinner and colleagues [12] indicated that conscientiousness was not an independent determinant of HbA_1c_, but behaviours that conscientiousness was associated with (such as not smoking, health BMI, and medication adherence), suggesting an indirect impact of conscientiousness of HbA_1c_.

Intelligence was also found to be associated with T2DM. For example, in a study Strachan et al [13] found that T2DM is significantly associated with decreased cognitive function in adults. In another study, Olsson, Hulting, and Montgomery [14] showed that lower cognitive function in children who would go on to have T2DM in adulthood [14].

The social gradient of physical health is well documented [15–16]. Many studies have shown the link between socioeconomic adversity and T2DM [17]. For example, Stringhini and colleagues [18] found the significant association of life-course socioeconomic status with chronic inflammation and T2DM risk.

However, few studies examined social, demographic, and the two individual differences variables intelligence and personality traits together. As these factors are inter-correlated, it is important to ascertain the influence of each type of factors in the prevalence of T2DM.

### Hypotheses

The current study looks at social and psychological correlates of T2DM. It concentrates on the role of personality traits on the self-report of T2DM in 50 year old adults together with childhood and adulthood social and psychological factors. From the previous literature we predicted that H1 higher level of parental social class is negatively associated the T2DM in adulthood; H2 higher childhood intelligence is negatively associated with the T2DM in adulthood; H1 parents or siblings had diabetes is positively associated with the T2DM in adulthood; and H4 neuroticism and conscientiousness would be inversely associated with the T2DM in adulthood.

## Method

### Sample

The National Child Development Study (the 1958 British birth cohort) is a large-scale longitudinal study of the 17,415 individuals who were born in Great Britain in a week in March 1958 [19]. The following analysis is based on data collected at birth, at ages 7, 11, 33 and at 50 years. Some 14,134 children at age 11 years completed tests of cognitive ability (response = 87%). At age 50 years, 8,532 participants completed a questionnaire on personality traits (response = 69%). Respondents also provided information on educational qualifications at age 33 years, occupational levels at age 50 years, and the prevalence of T2DM at age 50 years. The analytic sample comprises 5,032 cohort members (50% females) with complete data. Analysis of response bias in the cohort data showed that the achieved adult samples did not differ from their target sample across a number of critical variables (social class, parental education and sex), despite a slight under-representation of the most disadvantaged groups [20]. Bias due to attrition of the sample during childhood has been shown to be minimal [21–22].

### Measures

Childhood measures: Parental social class at birth was measured by the Registrar General′s measure of social class (RGSC). RGSC is defined according to occupational status and the associated education, prestige or lifestyle [23] and is assessed by the current or most recently held job. Where the father was absent, the social class (RGSC) of the mother was used. RGSC was coded on a six-point scale: I professional; II managerial/tech; IIIN skilled non-manual; IIIM skilled manual; IV semi-skilled; and V unskilled occupations [24]. Mothers also provided information on gestational age and birth weight, birth order, and whether parents and siblings of cohort members had diabetes. Childhood cognitive ability tests [25] were accessed when cohort members were at age 11 years consisting of 40 verbal and 40 non-verbal items and were administered at school. Adulthood measures: At age 33 years, participants were asked about their highest academic or vocational qualifications. Responses are coded to the six-point scale of National Vocational Qualifications levels (NVQ) which ranges from ‘none’ to ‘university degree/higher’/equivalent NVQ 5 or 6. Data on current or last occupation held by cohort members at age 50 were coded according to the Registrar General′s Classification of Occupations (RGSC) described above using a 6-point classification. Personality traits were assessed by the 50 questions from the International Personality Item Pool (IPIP) [26]. Responses (5-point, from “Strongly Agree” to “Strongly Disagree”) are summed to provide scores on the ‘Big-Five’ personality traits: Extraversion, Emotionality/Neuroticism, Conscientiousness, Agreeableness, and Intellect/Openness. At age 50 years 12,316 cohort members were invited for interviews and 9,790 were interviewed (79%), and answered the question of whether cohort member suffered from diabetes in last 12 months (Yes/No). As type 1 and type 2 diabetes are aetiologically distinct [27] we eliminated the majority of diagnoses of type 1 diabetes by excluding cohort members who had diabetes at age 16 year. Twenty-two cases were excluded from the study.

### Statistical Analysis

To investigate the correlates of T2DM in adulthood, first, *T*-test and ANOVA were conducted to examine the characteristics of the study population. Second, correlational analysis on the measures in the study were used. Following this we carried out a series of logistic regression analyses using STATA version 12 with T2DM at age 50 as the dependent variable. Three models were tested. Model 1 examines the associations between childhood factors and the outcome variable; Model 2 examines the associations between adult social factor and the outcome variable, together with the childhood factors in Model 1; Model 3 examines the associations between adult personality factors and the outcome variable, together with factors in Models 1 and 2. Gestational age and birth weight were controlled in all three models.

## Results

### Descriptive Analysis

Table 1 shows the characteristics of the study population according to the rate of T2DM at 50 years. It appears that the prevalence of T2DM was greater for men than for women (4.3% vs 2.5%). *T*-test showed that the differences of the rate of T2DM between men and women were statistically significant (*t* (*df* = 5030) = 3.49, *p* < .001).

**Table 1:** Social and demographic characteristics of the study population and prevalence of T2DM at age 50.

	n	%	Prevalence of T2DM %
*Sex*			
Male	2502	49.7	4.3
Female	2530	50.3	2.5
*Parental social class at birth*			
Unskilled (V)	355	8.7	4.2
Partly skilled (IV)	597	14.3	3.9
Skilled manual (III)	2468	54.1	3.8
Skilled non-manual (III)	564	7.0	2.1
Managerial/tech (II)	788	12.8	2.8
Professional (I)	260	4.1	2.7
*Educational qualifications at age 33*			
No qualifications	340	8.7	4.4
CSE 2–5/equivalent NVQ1	550	10.5	3.3
O Level/equivalent NVQ2	1717	37.8	3.8
A level/equivalent NVQ 3	818	13.4	2.8
Higher qualification/equivalent NVQ4	843	18.6	3.8
University Degree/equivalent NVQ 5, 6	764	11.0	2.5
*Own current social class at age 50*			
Unskilled (V)	104	1.2	1.9
Partly skilled (IV)	522	11.0	3.6
Skilled manual (III)	875	21.5	4.2
Skilled non-manual (III)	1052	21.5	3.5
Managerial/tech (II)	2163	39.5	3.1
Professional (I)	316	5.2	2.8

From Table 1 it shows that low parental and own social class (manual work) and no qualification had higher rates of the prevalence of T2DM.

Table 2 shows the correlations among the variables examined in the study.

**Table 2:** Pearson product-moment correlations of variables in the study.

	*Variables*	Mean (SD)	1	2	3	4	5	6	7	8	9	10	11
1.	T2DM at age 50	.03 (.18)	**_**										
2.	Sex	.50 (.50)	**-.049**	_									
3.	Parental social class	3.32(1.23)	**-.028**	-.019	_								
4.	Parents or siblings had diabetes	.02 (.15)	**.042**	.003	.002	_							
5.	Childhood intelligence	104.2 (12.8)	**-.028**	.081	.260	-.006	_						
6.	Educational qualifications	2.71 (1.44)	**-.020**	-.079	.313	-.002	.481	_					
7.	Own occupational levels	4.11 (1.20)	**-.013**	-.017	.204	.201	.323	.444	_				
8.	Extraversion α = .73	29.43 (6.60)	**-.027**	.074	.029	-.006	.021	.069	.120	_			
9.	Neuroticism α = .88	28.93 (7.02)	**-.021**	-.135	.026	-.025	.088	.087	.083	.208	_		
10.	Agreeableness α = .81	36.86 (5.27)	**-.032**	.401	.042	-.010	.115	.079	.101	.363	.054	_	
11.	Conscientiousness α = .77	34.02 (5.28)	**-.053**	.105	.019	.020	.039	.065	.093	.142	.192	.274	_
12.	Openness α = .79	32.57 (5.16)	**-.014**	-.019	.132	-.014	.276	.316	.239	.400	.102	.336	.223

*Note*: Variables were scored such that a higher score indicated being female, the presence of parents of siblings had diabetes, and T2DM in adulthood, a more professional occupation for parents or cohort members, higher scores on childhood intelligence, highest educational qualification, higher scores on traits extraversion, neuroticism, agreeableness, conscientiousness, and openness.

Table 2 shows that adulthood T2DM was significantly associated with sex, parental social class, childhood intelligence, parents or siblings had diabetes, trait agreeableness and trait conscientiousness (*p* < .05). Thus H1–H3 were confirmed, H4 was partially confirmed, as trait agreeableness, not trait neuroticism, that was significantly associated with the outcome variable.

Table 3 shows the odd ratios for T2DM at age 50, according to the occurrence of parental or sibling diabetes, childhood cognitive ability, educational qualifications, current occupational levels, and personality traits.

**Table 3:** Odds ratios (95% CI) for T2DM at age 50, according to the occurrence of parental or sibling diabetes, childhood cognitive ability, educational qualifications, current occupational levels, and personality traits.

	Model 1	Model 2	Model 3	
Model 1	Odds ratio (95% CI)	Odds ratio (95% CI)	Odds ratio (95% CI)	# *p*-value
*Childhood and family factors*				
Sex	0.61 (0.44, 0.85) **	0.56 (0.39, 0.80) **	0.57 (0.39, 0.84) **	0.004
Parental social class at birth (*unskilled as reference group*)				
Partly skilled	1.06 (0.52, 2.19)	1.07 (0.52, 2.21)	0.94 (0.46, 1.91)	0.857
Skilled manual	1.07 (0.57, 1.98)	1.07 (0.57, 1.98)	1.01 (0.55, 1.84)	0.980
Skilled non-manual	0.64 (0.28, 1.45)	1.07 (0.57, 1.99)	0.57 (0.25, 1.28)	0.173
Managerial/tech	0.76 (0.36, 1.59)	0.65 (0.28, 1.47)	0.80 (0.39, 1.64)	0.535
Professional	0.61 (0.21, 1.78)	0.79 (0.37, 1.68)	0.79 (0.30, 2.06)	0.624
Parents or siblings who had diabetes	2.64 (1.30, 5.34)**	2.69 (1.32, 5.46)**	2.79(1.36, 5.68)**	0.005
Childhood intelligence at age 11	0.91 (0.76, 1.08)	0.92 (0.75, 1.13)	0.91 (0.74, 1.11)	0.352
*Adult social factors*				
Educational qualifications (*no qualification as reference group*)				
CSE 2–5/equivalent NVQ1		0.57 (0.27, 1.21)	0.79 (0.37, 1.67)	0.531
O Level/equivalent NVQ2		0.90 (0.48, 1.65)	1.04 (0.55, 1.99)	0.895
A level/equivalent NVQ 3		0.59 (0.29, 1.23)	0.73 (0.35, 1.54)	0.415
Higher qualification/ equivalent NVQ4		0.94 (0.46, 1.90)	1.13 (0.54, 2.35)	0.741
University Degree/ equivalent NVQ 5, 6		0.64 (0.28, 1.48)	0.76 (0.33, 1.77)	0.526
Own social class (*unskilled as reference group*)				
Partly skilled		2.30 (0.52, 10.12)	2.08 (0.47, 9.22)	0.337
Skilled manual		1.84 (0.43, 7.88)	1.78 (0.41, 7.66)	0.440
Skilled non-manual		2.20 (0.51, 9.50)	2.33 (0.54, 10.06)	0.258
Managerial/tech		1.97 (0.46, 8.43)	1.93 (0.45, 8.29)	0.374
Professional		1.70 (0.34, 8.50)	1.76 (0.36, 8.70)	0.489
*Adult personality factors*				
Extraversion			0.91 (0.76, 1.10)	0.323
Neuroticism			0.94 (0.79, 1.12)	0.492
Agreeableness			1.00 (0.83, 1.21)	0.987
Conscientiousness			0.77 (0.66, 0.91)**	0.002
Openness			1.06 (0.87, 1.29)	0.554

*Note*:

**p < .01. Adjusted for gestational age and birth weight in all three models.

^#^ *p* values of the final model.

Table 3 shows that among childhood and family factors, parents or siblings was significantly association with the outcome variable. Cohort members whose parents or siblings had diabetes were more likely to have T2DM in adulthood, suggesting the familial (genetic and non-genetic) influence in this health problem. Among adult social and personality factors, trait conscientiousness was the only significant predictor of T2DM in adulthood. Sex was also significantly associated with the prevalence of T2DM. There was a greater prevalence of T2DM among men than among women.

## Discussion

This study set out to explore social and psychological factors that associated with the occurrence of T2DM in adulthood. Using a large, nationally representative population sample the current study provides some evidence of the associations between demographic, familial (genetic and non-genetic), and psychological factors and the occurrence of T2DM in adulthood.

Although previous studies in the area show that the T2DM is less strongly associated with familial and genetic factors [6, 28], and is more strongly influenced by behavioural, psychological, and social factors, such as eating behaviours and conscientious personality relating to self-control and discipline on diet, this study shows that the prevalence of T2DM in adulthood appears to be not free from genetic and non-genetic familial influences, Indeed among all the variables examined in the models, parents or siblings had diabetes are most strongly associated with T2DM, Cohort members who have parents or siblings suffering from diabetes are more than twice likely to have this health problem in adulthood.

On the other hand, this study shows that trait conscientiousness plays a role in influencing the occurrence of T2DM in adulthood, indicating that being careful in food selection and healthy diet may reduce the prevalence of T2DM in adulthood [2, 12, 28]. This reflects the findings of a recent study showing the link between the type of food intakes (anthocyanins and flavones) and biomarkers of insulin resistance [29]. None of the other personality factors had any effect. Indeed the literature on personality and health has shown as its most consistent finding that trait conscientiousness is the most common and robust predictor of health. Further, the size of the effect of trait conscientiousness is equal to or greater than that of many known biomedical risk factors. Studies that have looked at various illness have shown that conscientiousness predicts reduced disease development, better coping, and fewer symptoms.

The non-significant association between trait neuroticism and T2DM found in the study might be that many previous studies which did find the significant associations between these two measures used clinical samples rather than population based nationally representative samples with more extreme scores on neuroticism.

The current study also shows that higher parental social status and childhood intelligence are inversely associated with T2DM in adulthood (shown in correlational analysis) as found in some previous studies [13–14]. These associations are no longer significant once personality traits and familial influence are taken into account (shown in regression models), though the odd ratios of these factors are in the expected direction. This may in part, due to the length of interval between the measure of intelligence and T2DM. For example, although using the same dataset, the Olsson et al [13] study examined T2DM between 16 and 42 years of age and the current study looked at T2DM at 50 years, thus the time points between childhood intelligence (measured at 11 years) and the outcome variable in the current study is eight to thirty-four years longer than the previous study and this may decrease such association.

It is unclear why men have a greater prevalence of T2DM than women. This finding is in line with the results of the National Health Interview Survey 2004 in the US [30], which shows that in 2002, the age-adjusted incidence of diagnosed diabetes was 7.6 per 1000 for men and 6.7 per 1000 for women.

The results of the current study suggest that by understanding trait conscientiousness of patients with T2DM it might be possible to target interventions and provide support more effectively, and to encourage patients to manage their conditions better.

## Limitations

This study is based on a British cohort, and may not be representative internationally. Furthermore, this study is based on available variables in the dataset rather than being based on the study designed for the purpose, thus variables included in the study do not have a wide scope in investigating correlates of the outcome variable. Future studies are required to confirm the findings.
